# Supramodal Representation of the Sense of Body Ownership in the Human Parieto-Premotor and Extrastriate Cortices

**DOI:** 10.1523/ENEURO.0332-22.2023

**Published:** 2023-02-03

**Authors:** Yusuke Sonobe, Toyoki Yamagata, Huixiang Yang, Yusuke Haruki, Kenji Ogawa

**Affiliations:** Department of Psychology, Hokkaido University, Sapporo 060-0810, Japan

**Keywords:** functional magnetic resonance imaging (fMRI), multivoxel pattern analysis (MVPA), rubber hand illusion (RHI), sense of body ownership

## Abstract

The sense of body ownership, defined as the sensation that one’s body belongs to oneself, is a fundamental component of bodily self-consciousness. Several studies have shown the importance of multisensory integration for the emergence of the sense of body ownership, together with the involvement of the parieto-premotor and extrastriate cortices in bodily awareness. However, whether the sense of body ownership elicited by different sources of signal, especially visuotactile and visuomotor inputs, is represented by common neural patterns remains to be elucidated. We used functional magnetic resonance imaging (fMRI) to investigate the existence of neural correlates of the sense of body ownership independent of the sensory modalities. Participants received tactile stimulation or executed finger movements while given synchronous and asynchronous visual feedback of their hand. We used multivoxel patterns analysis (MVPA) to decode the synchronous and asynchronous conditions with cross-classification between two modalities: the classifier was first trained in the visuotactile sessions and then tested in the visuomotor sessions, and vice versa. Regions of interest (ROIs)-based and searchlight analyses revealed significant above-chance cross-classification accuracies in the bilateral intraparietal sulcus (IPS), the bilateral ventral premotor cortex (PMv), and the left extrastriate body area (EBA). Moreover, we observed a significant positive correlation between the cross-classification accuracy in the left PMv and the difference in subjective ratings of the sense of body ownership between the synchronous and asynchronous conditions. Our findings revealed the neural representations of the sense of body ownership in the IPS, PMv, and EBA that is invariant to the sensory modalities.

## Significance Statement

Previous studies have shown neural correlates of the sense of body ownership in parieto-premotor and extrastriate cortices. However, whether the sense of body ownership induced by different sensory inputs is represented in common neural patterns remains unelucidated. Using functional magnetic resonance imaging (fMRI) with multivoxel pattern analysis (MVPA), we investigated neural representations of the sense of body ownership invariant to modalities. Decoding neural patterns for visuotactile and visuomotor modalities revealed successful cross-classification accuracies in intraparietal sulcus (IPS), ventral premotor cortex (PMv), and extrastriate body area (EBA). Furthermore, cross-classification accuracy in PMv was positively correlated with subjective ratings of the sense of body ownership. These findings demonstrate that supramodal representations in parieto-premotor and extrastriate cortices underlie the sense of body ownership.

## Introduction

The brain receives multiple signals from the sensory channels. The integration of multimodal sensory information contributes to the coherent representation of the self (Tsakiris, 2010, 2017; Petkova et al., 2011; Blanke, 2012). Previous research in macaques has empirically revealed the involvement of the parieto-premotor cortices in the process of multisensorial information about the body vicinity. Neurons in the premotor and the parietal cortices are modulated by more than one sensory signal, and the integration is maximized when stimuli are spatiotemporally congruent. Considering that multisensory integration requires different sensory inputs to be unified in individual neurons, bimodal neurons are ideal candidates for multisensory convergence (Graziano et al., 1997; Duhamel et al., 1998; Avillac et al., 2007).

The sense of body ownership is a component of bodily self-consciousness based on multisensory integration and is defined as the sensation that part of or the whole body belongs to oneself. It has been approached by theoretical papers from both a phenomenological (Gallagher, 2000; Blanke and Metzinger, 2009) and a neurocognitive (Tsakiris, 2010; Blanke, 2012) perspective. Theoretical and empirical work has investigated the sense of body ownership, using the rubber hand illusion (RHI; Armel and Ramachandran, 2003; Tsakiris and Haggard, 2005; Apps and Tsakiris, 2014; Tsakiris, 2017). RHI is an experimental paradigm, whereby participants feel ownership of a fake hand placed in front of them after synchronous touch of their real hand and the fake one (Botvinick and Cohen, 1998). Converging evidence suggests that the illusion is induced when visual and proprioceptive signals are synchronized (Shimada et al., 2005, 2009, 2014; Bekrater-Bodmann et al., 2014).

RHI has been widely employed to investigate multisensory integration and the sense of body ownership albeit with methodological differences (for review, see Riemer et al., 2019). This might raise the issue of whether multisensory integration under different conditions induces the same perceptual experience. Although the findings are not consistent and depend on whether subjective ratings or proprioceptive drift were used, previous behavioral studies involving a direct comparison of visuotactile and visuomotor conditions reported that both conditions result in a similar ownership sensation (Tsakiris et al., 2006; Kalckert and Ehrsson, 2014a). However, whether the behaviorally similar sense of body ownership is represented by a common or by distinct spatial neural activities is unknown. Neuroimaging studies have identified brain regions associated with the sense of body ownership, such as the intraparietal sulcus (IPS), the ventral premotor cortex (PMv), and the extrastriate body area (EBA). However, the neural correlates of the sense of body ownership induced by tactile stimulation and kinesthetic movement have been investigated independently by different studies (Ehrsson et al., 2004; Tsakiris et al., 2010; Gentile et al., 2011; Olivé et al., 2015; Lee and Chae, 2016; Limanowski and Blankenburg, 2016a). Furthermore, prior functional magnetic resonance imaging (fMRI) studies have mainly conducted a univariate analysis of the overall activation increase in a region, not providing information about the similarities in the spatial activation patterns between different sensory modalities.

Here, we aimed to examine whether the sense of body ownership induced by visuotactile and visuomotor inputs is represented in common neural activation patterns. We manipulated the participant’s sense of ownership of their hand using tactile stimulation and finger movement within a single fMRI experiment. We then used multivoxel pattern analysis (MVPA). In contrast to the conventional subtraction-based fMRI analysis, MVPA enables the identification of differences in spatial patterns of activated voxels in a region (Haynes and Rees, 2005; Kamitani and Tong, 2005; Kriegeskorte et al., 2006; Norman et al., 2006). We thus examined whether differences in the multivoxel spatial patterns in synchronous and asynchronous conditions were common to each modality and predicted that the IPS, PMv, and EBA, regions associated with the sense of body ownership, could decode the differences regardless of the modalities.

## Materials and Methods

### Participants

Twenty-six healthy volunteers (14 males and 12 females) with a mean age of 22.38 years (20–28 years) participated in the experiment. The number of participants was determined based on previous fMRI experiments (Gentile et al., 2011; Bekrater-Bodmann et al., 2014). All participants were right-handed as assessed by a modified version of the Edinburgh Handedness Inventory (Oldfield, 1971). Written informed consents were obtained from all participants in accordance with the Declaration of Helsinki. The experimental protocol was approved by the local ethics committee. All participants were naive to the purpose of the fMRI experiment, although two of them had previously participated in behavioral RHI experiments. One participant with large head movements (maximum translation per session, 66.44 mm) during the scanning was excluded from the analysis. Thus, the final analysis included 25 participants.

### Experimental design and statistical analysis

We employed a two-by-two within-subject factorial design. We manipulated the type of modalities (i.e., visuotactile vs visuomotor) and the timings of the visual feedback (i.e., synchronous vs asynchronous). In the visuotactile condition, the participant’s right index finger was stroked with a paintbrush. In the visuomotor condition, the participants raised and lowered their right index finger on their own. In the synchronous condition, videos of the strokes or movements were presented in real-time. In the asynchronous condition, the videos were presented with a 1000-ms delay. The four conditions were labeled as visuotactile synchronous (TS), visuotactile asynchronous (TA), visuomotor synchronous (MS), and visuomotor asynchronous (MA).

Statistical analyses were performed using a two-way within-subject ANOVA, a one-sample *t* test, and a paired *t* test. To control for the problem of multiple comparisons, we applied the Holm–Bonferroni procedure (Holm, 1979) based on the number of regions of interest (ROIs) in the left and right hemispheres, respectively. We used Pearson’s *r* to investigate the correlation between the cross-classification accuracies and the subjective ratings in the questionnaire. The statistical significance level is reported for each analysis.

### Experimental setup

The participants lay comfortably in a supine position on the bed in the MRI scanner. The participant’s hand was extended on the bed in a relaxed position and hidden from their own view. A flexible arm (Articulated arm mount, MRC Systems GmbH) was attached to the head coil with an MRI-compatible color camera (12 M-i, MRC Systems GmbH) fixed at the end of the arm. This camera captured videos of the participant’s right hand. The videos were projected on the monitor using Light Capture (I-O DATA) in the control room in an anatomically congruent frame with respect to the real hand. A webcam (C270n, Logitech) captured the videos on the monitor using MATLAB (The MathWorks). The videos were presented on a liquid crystal display (NNL LCD Monitor, NordicNeuroLab) and projected onto a custom-made viewing screen. The participants viewed the screen via a mirror. Real-time video transfer was realized with a minimal intrinsic delay of ∼400 ms in the synchronous condition. The intrinsic delay refers to the time difference between the movement of the right hand and the movement of the hand on the screen. It was calculated by assessing how long (in ms) after the movement of the real hand, the movement of the hand appeared on the LCD using Adobe Premiere Pro (Adobe Inc.). In the asynchronous condition, a systematic delay of 1000 ms was added to the intrinsic delay. The distance between the camera and the real hand was adjusted before the experiment according to the size of the participant’s hand to make it easier for the participant to see their hand on the screen via a mirror. The video presentation was implemented by Computer Vision Toolbox (https://mathworks.com/products/computer-vision.html), Image Processing Toolbox (https://mathworks.com/products/image.html), MATLAB Support Package for USB Webcams (https://mathworks.com/matlabcentral/fileexchange/45182-matlab-support-package-for-usb-webcams), and Psychtoolbox (http://psychtoolbox.org/) for MATLAB.

**Figure 1.**
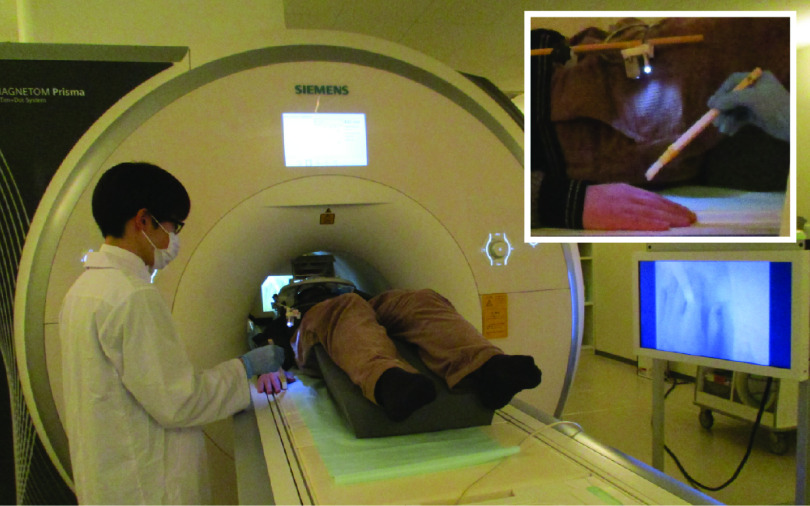
Experimental setup. The participants watched videos of their right hand captured by the camera attached to the end of the flexible arm on the head coil. In the visuotactile sessions, an experimenter stroked the participant’s right index finger at a frequency of ∼1 Hz to a beep sounded in the MRI room. In the visuomotor sessions, the participants lifted and lowered their right index finger on their own at the same rate to a beep heard through headphones while an experimenter stood beside them.

Participants heard a beep through MRI-compatible headphones (Resonance Technology Inc.) to ensure that the number of finger movements was the same among participants. They heard the beep both in the visuotactile and visuomotor sessions to prevent a confounding effect of the beep. The beep sound was created at a frequency of ∼1 Hz with a jitter of −200, 0, or +200 ms added pseudo-randomly to make the stimulation sequence unpredictable. The beep output from the computer was split in two: one output to the participant’s headphones and another sounded in the entire MRI room via the intercom as a cue for the experimenter to apply brushstrokes to the participant’s hand. The beep sounded in the entire MRI room both in the visuotactile and visuomotor sessions. The beep presentation was implemented by MATLAB (Fig. 1).

### Task procedures

There were four experimental sessions. The visuotactile and visuomotor sessions were each performed twice in succession. The order of sessions was pseudo-randomized across the participants. Each session consisted of 16 trials, eight in synchronous and eight in asynchronous conditions. Synchronous or asynchronous conditions were determined pseudo-randomly, but the same type of visual feedback was not repeated three times in succession.

**Figure 2.**
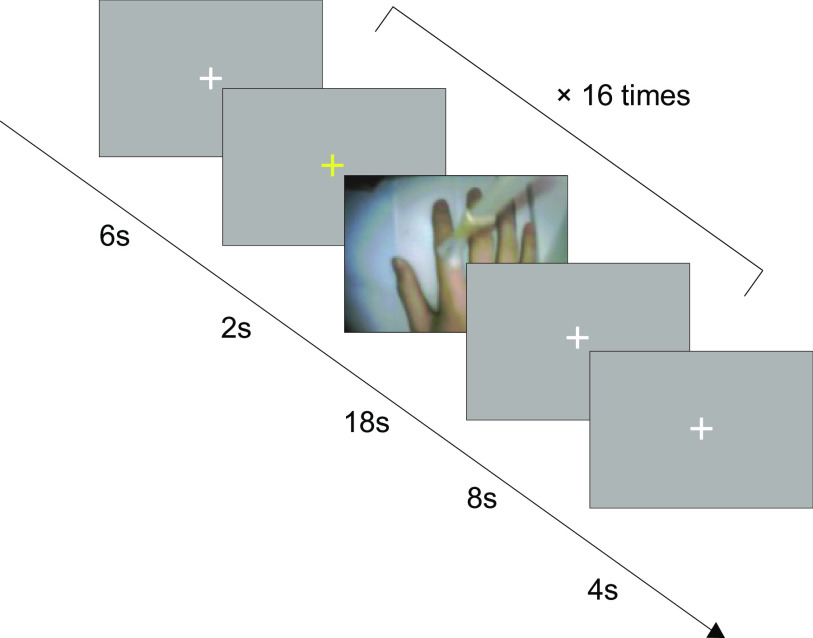
Schematic depiction of the time course of the visuotactile and visuomotor sessions. Trials began with the yellow fixation cross as the cue. Two seconds later, the videos of the right hand were displayed for 18 s, followed by the white fixation cross for 8 s. In eight out of 16 trials, the videos were presented synchronously with the tactile stimuli or finger movements. In the other eight trials, the videos were displayed with a systematic delay of 1000 ms. The videos were never synchronous or asynchronous three times in succession.

Each trial began with 2 s of yellow fixation cross, followed by 18 s of videos of the participant’s right hand. In the visuotactile sessions, tactile stimulation was delivered by an experimenter manually with a paintbrush at a frequency of ∼1 Hz during 18 s, as cued by a beep. The upper part of the participant’s right index finger was stroked from the fingertip to the knuckle at one beep and inversely from the knuckle to the fingertip at the next beep. To know when to start and end stroking, an experimenter checked the LCD placed adjacent to the MRI scanner during the stimulation. Because of a malfunction of the projector, the LCD was placed near the participant’s head for five subjects. The five participants viewed the videos on the LCD via a mirror. In these cases, an experimenter stroked the participant’s right index finger while looking inside the MRI scanner to check the LCD. In the visuomotor sessions, the participants lifted their right index finger at one beep and lowered it at the next beep at the same rate during 18 s. To remove the confounding influence of the presence of people between the visuotactile and visuomotor sessions, an experimenter kept standing beside the participant in the same position as in the visuotactile sessions. Subsequently, a white fixation cross was presented for 8 s and served as baseline (Fig. 2). The participants heard a beep via MRI-compatible headphones throughout the fMRI scanning period. The videos were recorded and analyzed after the experiment. The average (SD) number of brush strokes and finger movements for each participant was 16.55 (0.95). The participants practiced the finger movements for 1 min before they entered the MRI scanner.

After every two sessions, the participants completed questionnaires to report their subjective experience in the MRI scanner (Table 1). The questionnaire items were based on those used by Kalckert and Ehrsson (2014a,b). The questionnaire consisted of six items with items (1)–(4) serving as indicators of the sense of body ownership (Ownership) and with items (5) and (6) serving as controls for ownership, in particular for task compliance, suggestibility, and expectancy effects (Ownership control; Kalckert and Ehrsson, 2014a,b). The item order was pseudo-randomized. Responses were self-paced and were made using a seven-point Likert scale, ranging from +3 (strongly agree) to −3 (strongly disagree), with 0 indicating neither agreement nor disagreement or “uncertainty.” We recorded the responses using MRI-compatible response pads (Current Design). The participants answered the six items twice in succession, once for the synchronous condition and once for the asynchronous condition. The order of questionnaire items was pseudo-randomized across the participants, but with the same order across the modalities within a participant. To avoid the confounding effect of the hands used to rate items, the participants were pseudo-randomly divided into two groups according to which hand they used to rate items as positive. The right index finger, which was stimulated by the paintbrush and used for finger movements, was not assigned to press the button. The questionnaire presentation was implemented by PsychoPy (Peirce et al., 2019).

**Table 1:** Statements used in the questionnaire to quantify the subjective experience of the sense of body ownership

Ownership	1	I felt as if I was looking at my own hand
	2	I felt as if the hand in the video was part of my body
	3	I felt as if the hand in the video was my hand
	4	I felt as if my real hand was turning the hand into the video
Ownership control	5	It seems as if I had more than one right hand
	6	It felt as if I had no longer a right hand, as if my right hand had disappeared

As the present study did not use a fake hand but presented their own hand to the participants via video feedback, the item “I felt as if my real hand was turning the hand into the video” adapted from Kalckert and Ehrsson (2014a,b) was analyzed as Ownership.

### Functional EBA localizer scan

An independent functional localizer scan was performed for all participants after the visuotactile and visuomotor sessions to determine a ROI of the EBA selectively activated by body parts (Downing et al., 2001). The protocol was adapted from a previous study (Taylor et al., 2007). A total of 16 blocks were performed, which consisted of 8 body blocks and 8 control blocks. A body block consisted of 20 images of headless human body parts in different postures, which were alternated with a control block consisting of 20 images of chairs. All images were grayscale. Each image was presented for 300 ms, followed by a black screen for 450 ms. A fixation cross was intercalated at the end of each block for 12 s as baseline. To maintain and monitor attention, the participants performed a one-back repetition detection task during the scan. The same images were presented twice in succession, twice during each block. The participants were asked to press a button with the right index finger when they detected the immediate repetitions. The responses were recorded using MRI-compatible response pads. The participants practiced a shorter version of the task outside the MRI scanner before the experiment. The stimuli presentation was implemented by PsychoPy (Peirce et al., 2019).

### MRI data acquisition

All scans were performed on a Siemens 3-Tesla Prisma scanner with a 20-channel head coil at Hokkaido University. T2*-weighted echoplanar imaging (EPI) was used to acquire a total of 229 and 219 scans per session for the main and localizer sessions, respectively, with a gradient echo EPI sequence. The first three scans of each session were discarded to allow for T1 equilibration. The scanning parameters were repetition time (TR), 2000 ms; echo time (TE), 30 ms; flip angle (FA), 90°; field of view (FOV), 192 × 192 mm; matrix, 94 × 94; 32 axial slices; and slice thickness, 3.5 mm with a 0.875 mm gap. T1-weighted anatomic imaging with an MP-RAGE sequence was performed using the following parameters TR, 2300 ms; TE, 2.32 ms; FA, 8°; FOV, 240 × 240 mm; matrix, 256 × 256; 192 axial slices; and slice thickness, 0.90 mm without a gap.

### Processing of fMRI data

Image preprocessing was performed using the SPM12 software (Wellcome Department of Cognitive Neurology, http://www.fil.ion.ucl.ac.uk/spm). All functional images were initially realigned to adjust for motion-related artifacts. Volume-based realignment was performed by co-registering images using rigid-body transformation to minimize the squared differences between volumes. The realigned images were then spatially normalized with the Montreal Neurologic Institute (MNI) template based on the affine and nonlinear registration of co-registered T1-weighted anatomic images (normalization procedure of SPM). They were resampled into 3 × 3 × 3 mm voxels with sinc interpolation. Images were spatially smoothed using a Gaussian kernel of 6 × 6 × 6 mm full width at half-maximum. However, images used for MVPA were not smoothed to avoid blurring the fine-grained information contained in the multivoxel activity (Mur et al., 2009; Kamitani and Sawahata, 2010).

Using the general linear model, the 16 blocks per session were modeled as 16 separate boxcar regressors that were convolved with a canonical hemodynamic response function. Low-frequency noise was removed using a high-pass filter with a cutoff period of 128 s, and serial correlations among scans were estimated with an autoregressive model implemented in SPM12. This analysis yielded 16 independently estimated parameters (β values) per session for each individual voxel. These parameter estimates were then *z*-normalized across voxels for each trial and were subsequently used as inputs to MVPA.

### Definition of ROIs

We chose the IPS and PMv as ROIs because previous studies revealed that these regions are primarily involved in the multisensory processes implicated in the sense of body ownership. Electrophysiological studies reported that neurons located in the premotor and parietal cortices of macaques respond to stimuli from not only one but also multiple sensory modalities (Graziano et al., 1997; Duhamel et al., 1998; Avillac et al., 2007). This finding is supported by neuroimaging research in humans revealing greater activations in the IPS and PMv when both visual and tactile stimuli are presented compared with the activation resulting from either a visual or a tactile stimulus (Gentile et al., 2011). Studies using RHI consistently found that multisensory integration occurs in the IPS and PMv when stimuli are spatiotemporally congruent, resulting in the self-attribution of a fake hand (Ehrsson et al., 2004; Limanowski and Blankenburg, 2015; Olivé et al., 2015). Recent studies using transcranial magnetic stimulation (TMS) and transcranial direct current stimulation (tDCS) also revealed the involvement of these regions in the sense of body ownership (Karabanov et al., 2017; Convento et al., 2018; Lira et al., 2018). The finding is confirmed by a meta-analysis of human neuroimaging studies that investigated the relationship between peripersonal space and the sense of body ownership (Grivaz et al., 2017). Grivaz et al. (2017) have shown that the posterior parietal cortex (the bilateral IPS and superior parietal lobule), right PMv, and left anterior insula are involved in the sense of body ownership. Therefore, the IPS and PMv are likely responsible for the multisensorial process of body ownership.

Additionally to the frontoparietal regions, the EBA, which is part of the lateral occipital cortex (LOC), was chosen as ROI. The EBA has been associated with the visual processing of parts of or all the human body by Downing et al. (2001). However, recent neuroimaging studies have shown the involvement of the EBA in the sense of body ownership. For example, Limanowski and Blankenburg (2015) revealed increased connectivity between the LOC and IPS during RHI. The EBA activity is positively correlated with subjective illusion scores (Limanowski et al., 2014). Wold et al. (2014) reported an increased proprioceptive drift toward the rubber hand after rTMS of the left EBA.

**Figure 3.**
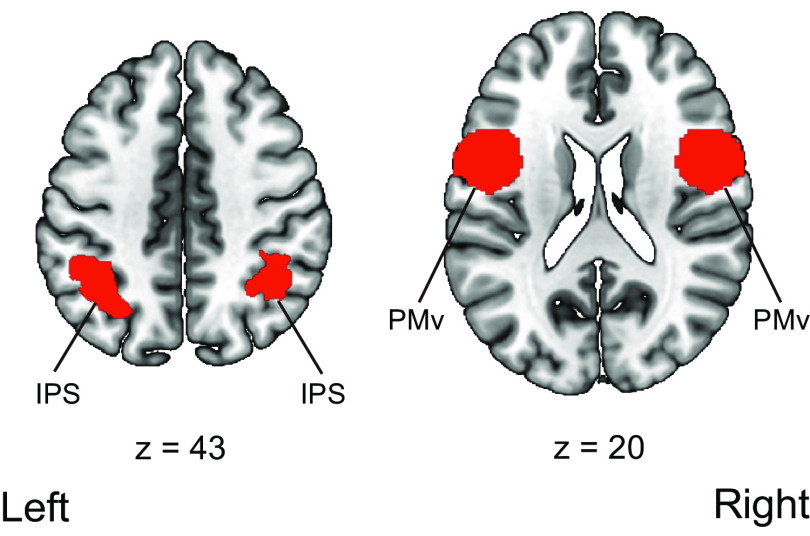
ROIs used in the univariate analysis of the parameter estimates and ROI-based multivoxel pattern analysis. The bilateral intraparietal sulcus was defined using Anatomy 13 toolbox (Eickhoff et al., 2005). The bilateral ventral premotor cortex was defined using the Human Motor Area Template 16 (Laboratory for Rehabilitation Neuroscience, http://lrnlab.org/; Mayka et al., 2006). IPS, intraparietal sulcus; PMv, ventral premotor cortex.

We followed the previous studies’ procedures as to whether we used a localizer task to define the IPS, PMv, and EBA. Previous human neuroimaging studies for the sense of body ownership have used no specific functional localizer task to define the IPS and PMv (Gentile et al., 2013; Limanowski and Blankenburg, 2015, [Bibr B39][Bibr B40], 2018; Lee and Chae, 2016). Therefore, anatomic templates for the IPS and PMv were used. The bilateral IPS was defined using Anatomy toolbox (Eickhoff et al., 2005), and the bilateral PMv was defined using the Human Motor Area Template (Laboratory for Rehabilitation Neuroscience, http://lrnlab.org/; Mayka et al., 2006; Fig. 3).

Conversely, the EBA cannot be identified using templates based on anatomic structures. The prior studies have used a localizer task to determine the EBA (Limanowski et al., 2014; Olivé et al., 2015; Limanowski and Blankenburg, 2016b). Following the procedure used by Olivé et al. (2015), we measured a significant EBA activation during the presentation of body part images compared with that elicited by chair images and determined a sphere with a radius of 5 mm centered on MNI coordinates of the group-level analysis with a threshold of *p *<* *0.05 corrected for family-wise error (FWE) with an extent threshold of 10 voxels (MNI coordinates: [−48, −70, 8] for the left hemisphere and [51, −58, 2] for the right hemisphere).

### Mass-univariate analysis

We used the conventional mass-univariate analysis of individual voxels to reveal areas activated in each condition and its combination. First, we analyzed the main effect of modalities collapsed across timings of visual feedback, [(TS + TA) − (MS + MA)] and inversely [(MS + MA) − (TS + TA)]. Second, we analyzed the main effect of timings of visual feedback collapsed across modalities, [(TS + MS) − (TA + MA)] and inversely [(TA + MA) − (TS + MS)]. Third, we analyzed the interaction effects between modalities and timings of visual feedback, [(TS−TA) − (MS−MA)] and inversely [(MS−MA) − (TS−TA)]. For the analysis of the EBA localizer scan, we compared areas activated during the presentation of body parts with regions activated by chair pictures.

Contrast images were generated for each participant using a fixed-effects model and were analyzed using a random-effects model of a one-sample *t* test. Activation was reported with a threshold of *p *<* *0.05 corrected for FWE at the voxel-level with an extent threshold of 10 voxels. If no area survived the threshold, activations with a threshold of *p *<* *0.001 uncorrected for multiple comparisons at the voxel-level with an extent threshold of 10 voxels were reported. The brain region names were reported with reference to the automated anatomic labeling atlas 3 (AAL3; Rolls et al., 2020). We additionally compared the averaged parameter estimates (β values) of ROIs using a two-way within-subject ANOVA with modalities (i.e., visuotactile vs visuomotor) and timings of visual feedback (i.e., synchronous vs asynchronous) as factors.

### Multivoxel pattern analysis (MVPA)

The multivariate classification analysis of fMRI data was performed with a multiclass classifier based on a linear support vector machine implemented in LIBSVM (http://www.csie.ntu.edu.tw/∼cjlin/libsvm/) with default parameters (a fixed regularization parameter C = 1). Multiclass classification, implemented in LIBSVM, was used to classify the representations of synchronous and asynchronous conditions. Parameter estimates (β values) of each trial of voxels within ROIs were used as inputs to the classifier.

We performed two within-modality and one cross-modality classification analyses. First, we ran the within-modality classification analyses between two visuotactile sessions and between two visuomotor sessions. The classifier was first trained to discriminate the representations of the synchronous and asynchronous conditions in the first visuotactile session. The same decoder was then tested to assess whether it could classify the representations of the synchronous and asynchronous conditions in the second visuotactile session. We also conducted a classification in the reverse direction: trained in the second visuotactile session and tested in the first visuotactile session. The averaged decoding accuracy was estimated. The same processing procedure was applied to the within-visuomotor classification analysis. Second, we performed a cross-modality classification analysis between the visuotactile and visuomotor sessions. The classifier was first trained to discriminate between the synchronous and asynchronous conditions in the visuotactile sessions. The same decoder was then tested to determine whether it could classify the representations of the synchronous and asynchronous conditions in the visuomotor sessions. We also conducted a classification in the reverse direction: trained in the visuomotor sessions and tested in the visuotactile sessions. The averaged decoding accuracy was estimated. Such a cross-conditional MVPA or cross-classification, a cross-validation between trials with different sets of tasks or stimuli, has been previously used to investigate the similarity or invariance of neural representations by testing the generalization of a classifier between different conditions or modalities (Kaplan et al., 2015). A one-sample *t* test was used to determine whether the observed decoding accuracy was significantly higher than chance (50%) with intersubject difference treated as a random factor (df = 24). For a ROI-based MVPA, we applied the Holm–Bonferroni procedure (Holm, 1979) based on the number of ROIs in the left and right hemispheres, respectively to control for the problem of multiple comparisons.

Complementary to the a priori ROI analysis, we additionally conducted a volume-based “searchlight” analysis (Kriegeskorte et al., 2006). The classification was performed using multivoxel activation patterns within a 9 mm-radius sphere (searchlight) that contained 123 voxels. The searchlight moved over the gray matter of the whole brain. The average classification accuracy for each searchlight with cross-validation was assigned to the sphere’s center voxel. The resulting map of the decoding accuracy was averaged over the participants. We used an uncorrected threshold of *p *<* *0.001 at the voxel-level and a threshold of *p *<* *0.05 FWE corrected at the cluster-level for each type of classification analysis. The names of the brain regions are reported with reference to the AAL3 (Rolls et al., 2020).

## Results

### Questionnaire subjective ratings

The behavioral ratings showed a stronger sense of body ownership in the synchronous condition than in the asynchronous condition. The mean ratings for items (1)–(4) were above 0 in the visuotactile and visuomotor synchronous conditions [visuotactile synchronous condition: (1) 1.16, SD ±1.64; (2) 1.20, SD ±2.09; (3) 1.16, SD ±2.17; (4) 0.68, SD ±1.92 and visuomotor synchronous condition: (1) 0.52, SD ±1.97; (2) 0.76, SD ±2.14; (3) 0.64, SD ±2.09; (4) 0.04, SD ±1.76], demonstrating that the participants felt as if the hand on the screen was their own in the synchronous condition. In contrast, the mean ratings for items (5) and (6) were below 0 in the visuotactile and visuomotor asynchronous conditions [visuotactile asynchronous condition: (5) −1.40, SD ±1.74; (6) −2.04, SD ±1.18 and visuomotor asynchronous condition: (5) −1.64, SD ±1.81; (6) −1.24, SD ±2.10], indicating that the participant’s feeling of ownership for the hand on the screen was less strong than that in the synchronous condition.

**Figure 4.**
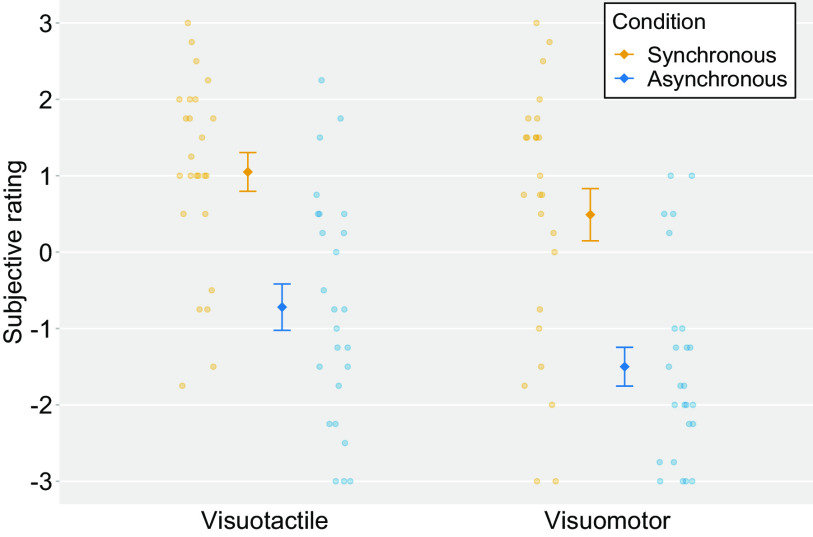
Mean ratings for Ownership questions in four conditions (ownership score). The light-colored circles correspond to the raw data of each participant (*n *=* *25) and the means are represented as dark-colored markers. Error bars indicate the SEM.

Next, the ratings for items (1)–(4) for each condition were averaged to obtain an ownership score and were analyzed by a two-way within-subject ANOVA with modalities (i.e., visuotactile vs visuomotor) and timings of visual feedback (i.e., synchronous vs asynchronous) as factors. We found a significant main effect of modalities (*F*_(1,24)_ = 7.22, *p *=* *0.01, *η^2^_p_* = 0.23). The mean ratings were higher in the visuotactile condition than those in the visuomotor condition. We also observed a significant main effect of timings of visual feedback (*F*_(1,24)_ = 51.12, *p *<* *0.01, *η^2^_p_* = 0.68), and the mean ratings were higher in the synchronous condition than they were in the asynchronous condition. There was no significant interaction effect between modalities and timings of visual feedback (*F*_(1,24)_ = 0.72, *p *=* *0.40, *η^2^_p_* = 0.03; Fig. 4).

Lastly, we conducted a paired *t* test to compare the individual questionnaire items between the synchronous and asynchronous conditions. There were statistically significantor statistical trend toward significant differences [visuotactile synchronous condition vs visuotactile asynchronous condition: (1) *t *=* *5.11, df* *=* *24, *p *<* *0.01; (2) *t *=* *6.18, df* *=* *24, *p *<* *0.01; (3) *t *=* *4.11, df* *=* *24, *p *<* *0.01; (4) *t *=* *4.13, df* *=* *24, *p *<* *0.01; (5) *t* = −2.22, df* *=* *24, *p *=* *0.04; (6) *t* = −1.74, df* *=* *24, *p *=* *0.09; visuomotor synchronous condition vs visuomotor asynchronous condition: (1) *t *=* *5.02, df* *=* *24, *p *<* *0.01; (2) *t *=* *6.72, df* *=* *24, *p *<* *0.01; (3) *t *=* *5.77, df* *=* *24, *p *<* *0.01; (4) *t *=* *5.48, df* *=* *24, *p *<* *0.01; (6) *t* = −3.15, df* *=* *24, *p *<* *0.01]. The ratings were higher in the synchronous condition than those in the asynchronous condition for items (1)–(4).

### Mass-univariate analysis

**Figure 5.**
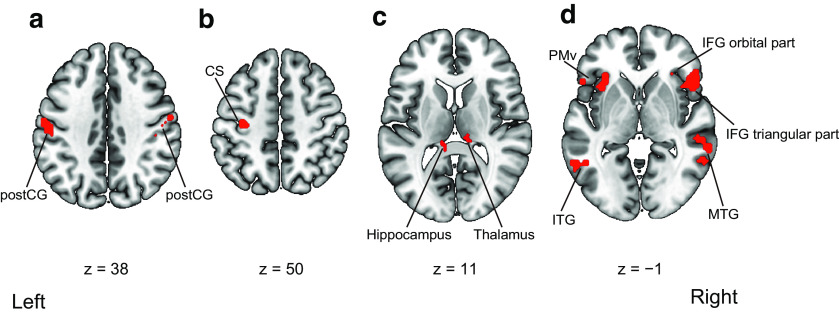
Regions activated by the main effects of modalities and timings of visual feedback in the fMRI univariate analysis. ***a***, Activated regions in the visuotactile condition compared with the visuomotor condition. ***b***, Activated regions in the visuomotor condition compared with the visuotactile condition. ***c***, Activated regions in the synchronous condition compared with the asynchronous condition. ***d***, Activated regions in the asynchronous condition compared with the synchronous condition. For the main effect of visuotactile and visuomotor conditions, activation was reported with a threshold of *p *<* *0.05 corrected for family-wise error (FWE) with an extent threshold of 10 voxels. For the main effect of synchronous and asynchronous conditions, activation was reported with a threshold of *p *<* *0.001 uncorrected for multiple comparisons with an extent threshold of 10 voxels. Montreal Neurologic Institute (MNI) coordinates of the activated foci are reported in Tables 2 and 3. postCG, postcentral gyrus; CS, central sulcus; ITG, inferior temporal gyrus; PMv, ventral premotor cortex; IFG, inferior frontal gyrus; MTG, middle temporal gyrus.

We first compared the activities in the visuotactile and visuomotor conditions collapsed across timings of visual feedback. The activations in the left inferior temporal gyrus, the bilateral postcentral gyrus, and the bilateral rolandic operculum were significantly greater in the visuotactile condition than those in the visuomotor condition. In contrast, the left central sulcus was more activated in the visuomotor condition than in the visuotactile condition. Next, we compared the activities in the synchronous and asynchronous conditions collapsed across modalities. We observed greater activations in the left hippocampus and the right thalamus in the synchronous condition than those in the asynchronous condition. The right triangular part of the inferior frontal gyrus, the left PMv, and the right middle temporal gyrus (MTG) were more activated in the asynchronous condition than in the synchronous condition. The anatomic locations of the activated regions are reported in Tables 2 and 3 and Figure 5.

**Table 2:** Anatomical regions, peak voxel coordinates, and *t* values of observed activation for the main effect of the visuotactile and visuomotor conditions

Anatomic region	Voxels	MNI coordinates			*t* value
		*x*	*y*	*z*	
Visuotactile > visuomotor					
L inferior temporal gyrus	101	−48	−67	−7	11.94
L middle occipital gyrus		−45	−70	2	8.66
L middle occipital gyrus		−39	−79	8	6.55
L postcentral gyrus	226	−54	−16	38	11.20
L postcentral gyrus		−27	−37	59	10.20
L postcentral sulcus		−45	−28	47	9.88
R Rolandic operculum	57	45	−19	17	11.19
L Rolandic operculum	130	−45	−19	20	10.99
L Heschl’s gyrus		−39	−16	11	9.43
L insula		−39	−10	5	9.31
R postcentral gyrus	23	57	−10	35	8.47
R postcentral gyrus		60	−13	44	7.51
R postcentral gyrus		51	−16	38	6.39
R postcentral gyrus	13	45	−25	50	8.23
R postcentral gyrus		45	−22	41	6.87
L superior temporal gyrus	10	−60	−4	8	7.69
L superior occipital gyrus	18	−24	−76	29	7.02
L superior occipital gyrus		−21	−85	23	6.90
Visuomotor > visuotactile					
L central sulcus	32	−33	−19	50	8.90
L central sulcus		−30	−25	59	7.14

Activation was reported with a threshold of *p *<* *0.05 corrected for family-wise error (FWE) with an extent threshold of 10 voxels. MNI, Montreal Neurologic Institute; L, left hemisphere; R, right hemisphere.

**Table 3:** Anatomical regions, peak voxel coordinates, and *t* values of observed activation for the main effect of synchronous and asynchronous conditions

Anatomic region	Voxels	MNI coordinates			*t* value
		*x*	*y*	*z*	
Synchronous > asynchronous					
L hippocampus	15	−12	−34	11	4.56
R thalamus	14	12	−28	11	4.43
Asynchronous > synchronous					
R inferior frontal gyrus, triangular part	731	51	26	−1	6.43
R ventral premotor cortex		39	5	29	6.27
R middle frontal gyrus		30	5	50	5.77
L inferior temporal gyrus	235	−60	−58	−4	6.37
L middle temporal gyrus		−48	−49	14	4.93
L inferior parietal lobule		−57	−40	44	4.77
L ventral premotor cortex	319	−33	5	29	6.31
L ventral premotor cortex		−51	17	38	5.76
L inferior frontal gyrus, triangular part		−57	23	8	4.94
R supplementary motor area	107	6	23	59	6.07
R superior frontal gyrus, dorsolateral		15	14	53	4.95
L supplementary motor area		−6	14	50	4.69
R middle temporal gyrus	465	54	−49	17	5.83
R middle temporal gyrus		57	−16	−10	5.12
R middle temporal gyrus		60	−37	−7	5.01
L intraparietal sulcus	46	−30	−58	44	5.68
R precuneus	44	9	−61	53	5.30
R precuneus		6	−52	41	4.10
R inferior frontal gyrus, orbital part	17	42	38	−13	5.19
R inferior frontal gyrus, orbital part		33	38	−13	3.80
R intraparietal sulcus	139	42	−58	56	4.79
R intraparietal sulcus		42	−55	47	4.48
L middle frontal gyrus	33	−30	2	56	4.51
R inferior frontal gyrus, orbital part	16	33	26	−7	4.44
R middle frontal gyrus	31	33	56	14	4.42
R middle frontal gyrus		36	59	5	3.85
L crus I of cerebellar hemisphere	11	−21	−79	−25	4.36
L crus II of cerebellar hemisphere		−12	−79	−34	3.86

Activation was reported with a threshold of *p *<* *0.001 uncorrected for multiple comparisons with an extent threshold of 10 voxels. MNI, Montreal Neurologic Institute; L, left hemisphere; R, right hemisphere.

**Figure 6.**
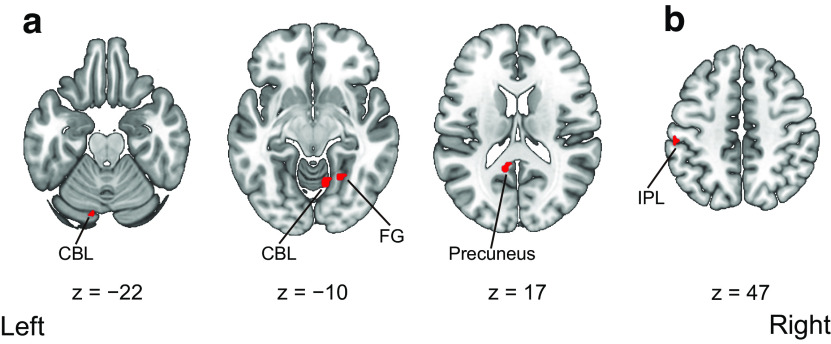
Regions activated by the interactions of modalities and timings of visual feedback in the fMRI univariate analysis. ***a***, Activated regions that showed the greater difference between the visuotactile synchronous and asynchronous conditions compared with the difference between the visuomotor synchronous and asynchronous conditions. ***b***, Activated regions that showed the greater difference between the visuomotor synchronous and asynchronous conditions compared with the difference between the visuotactile synchronous and asynchronous conditions. Activation was reported with a threshold of *p *<* *0.001 uncorrected for multiple comparisons with an extent threshold of 10 voxels. Montreal Neurologic Institute (MNI) coordinates of the activated foci are reported in Table 4. CBL, cerebellum; FG, fusiform gyrus; IPL, inferior parietal lobule.

We then investigated the areas showing interaction effects between modalities and timings of visual feedback. In the visuotactile condition, the difference between synchronous and asynchronous conditions was significantly larger in the left precuneus, the bilateral cerebellum, and the right fusiform gyrus than that in the visuomotor condition. In the visuomotor condition, the difference between the synchronous and asynchronous conditions was significantly greater in the left inferior parietal lobule than that in the visuotactile condition. The anatomic locations of the activated areas are reported in Table 4 and Figure 6.

**Table 4:** Anatomical regions, peak voxel coordinates, and *t* values of observed activation for the interaction effects

Anatomic region	Voxels	MNI coordinates			*t* value
		*x*	*y*	*z*	
Visuotactile synchronous interaction					
L precuneus	12	−9	−49	17	4.70
L crus I of cerebellar hemisphere	11	−12	−82	−22	4.05
R lobule VI of cerebellar hemisphere	16	12	−61	−10	4.03
R lingual gyrus		18	−67	−4	3.72
R fusiform gyrus	13	24	−55	−10	3.96
Visuomotor synchronous interaction					
L inferior parietal lobule	20	−57	−28	47	4.99
L inferior parietal lobule		−54	−31	38	3.82

Activation was reported with a threshold of *p *<* *0.001 uncorrected for multiple comparisons with an extent threshold of 10 voxels. MNI, Montreal Neurologic Institute; L, left hemisphere; R, right hemisphere.

**Figure 7.**
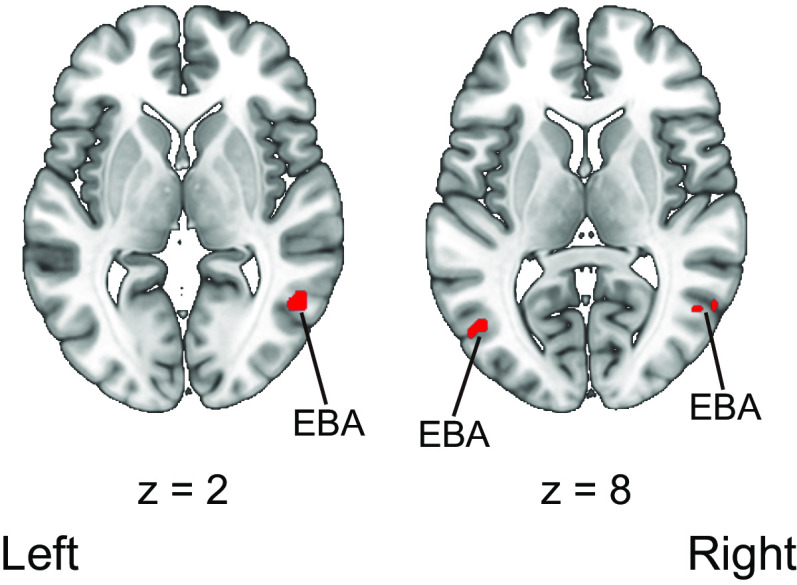
Regions activated by the presentation of body parts images compared with that of chair images during the EBA localizer scan. Activation was reported with a threshold of *p *<* *0.05 corrected for family-wise error (FWE) with an extent threshold of 10 voxels. Montreal Neurologic Institute (MNI) coordinates of the activated foci are reported in Table 5. EBA, extrastriate body area.

The fMRI data analysis of the EBA localizer scan revealed a significant activation of the left middle occipital gyrus and the right MTG when the participants watched body parts pictures compared with the activity elicited by chair images (MNI coordinates: [−48, −70, 8] for the left hemisphere and [51, −58, 2] for the right hemisphere). The anatomic locations of the activated areas are indicated in Table 5 and Figure 7. We also assessed the number of correct responses, namely pressing the button when the same images appeared twice successively in the body part and chair image blocks, to confirm whether the participants watched the stimulus. The results showed that the percentage of correct responses was 97.6%, indicating that the participants watched the stimulus closely.

**Table 5:** Anatomical regions, peak voxel coordinates, and *t* values of observed activation during the EBA localizer scan

Anatomic region	Voxels	MNI coordinates			*t* value
		*x*	*y*	*z*	
Body parts > chairs					
L middle occipital gyrus (EBA)	10	−48	−70	8	8.31
R middle temporal gyrus (EBA)	31	51	−58	2	8.09
R middle temporal gyrus (EBA)		51	−61	11	7.73

Activation was reported with a threshold of *p *<* *0.05 corrected for family-wise error (FWE) with an extent threshold of 10 voxels. MNI, Montreal Neurologic Institute; L, left hemisphere; R, right hemisphere.

Next, we extracted the parameter estimates of ROIs for each condition. The averaged parameter estimates (β values) were analyzed using a two-way within-subject ANOVA with modalities (i.e., visuotactile vs visuomotor) and timings of visual feedback (i.e., synchronous vs asynchronous) as factors. There was a significant main effect of the modalities in the bilateral IPS (*F*_(1,24)_ = 9.32, *p *=* *0.01, *η^2^_p_* = 0.28 for the left hemisphere and *F*_(1,24)_ = 7.47, *p *=* *0.01, *η^2^_p_* = 0.24 for the right hemisphere), the bilateral PMv (*F*_(1,24)_ = 8.77, *p *=* *0.01, *η^2^_p_* = 0.27 for the left hemisphere and *F*_(1,24)_ = 6.19, *p *=* *0.02, *η^2^_p_* = 0.21 for the right hemisphere), and the left EBA (*F*_(1,24)_ = 46.99, *p *<* *0.01, *η^2^_p_* = 0.66). The averaged parameter estimates were greater in the visuotactile condition than those in the visuomotor condition.

**Figure 8.**
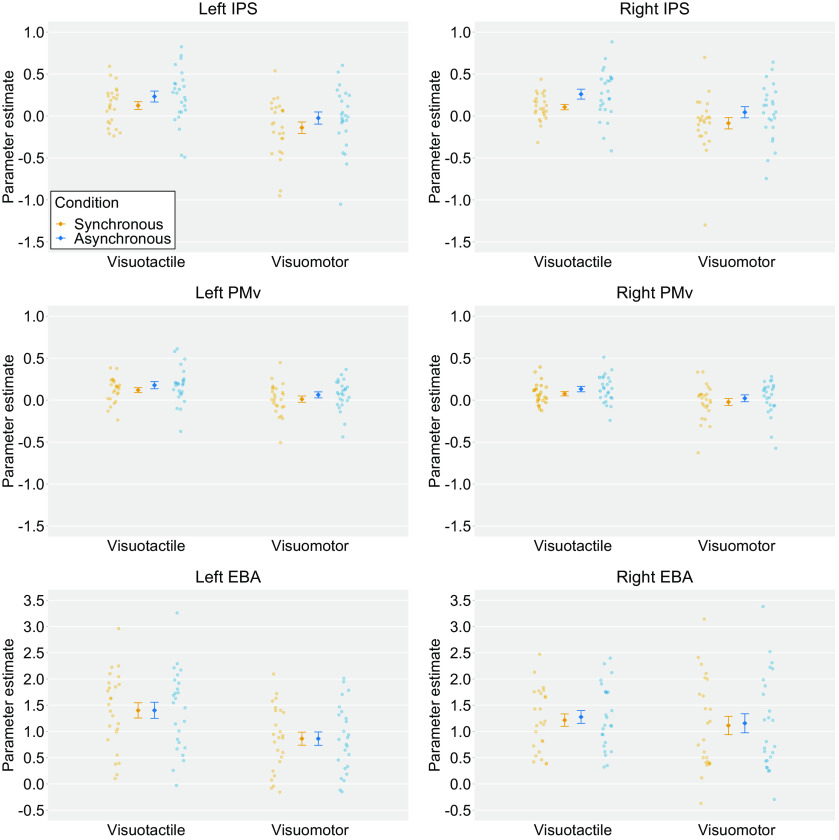
Averaged activation (parameter estimates) within ROIs. The light-colored circles correspond to the raw data of each participant (*n *=* *25) and the means are represented as dark-colored markers. Error bars indicate SEM. IPS, intraparietal sulcus; PMv, ventral premotor cortex; EBA, extrastriate body area.

We also found a significant main effect of the timings of the visual feedback in the bilateral IPS (*F*_(1,24)_ = 9.65, *p *<* *0.01, *η^2^_p_* = 0.29 for the left hemisphere and *F*_(1,24)_ = 13.65, *p *<* *0.01, *η^2^_p_* = 0.36 for the right hemisphere) and the bilateral PMv (*F*_(1,24)_ = 10.16, *p *<* *0.01, *η^2^_p_* = 0.30 for the left hemisphere and *F*_(1,24)_ = 8.51, *p *=* *0.01, *η^2^_p_* = 0.26 for the right hemisphere). There was a statistical trend toward a main effect of the timings of the visual feedback for the right EBA (*F*_(1,24)_ = 3.21, *p *= 0.09, *η^2^_p_* = 0.12). The parameter estimates in the aforementioned ROIs were significantly stronger in the asynchronous condition than those in the synchronous condition. No significant interaction between modalities and timings of visual feedback was observed in all ROIs (*F*s_(1,24)_ < 0.14, *p*s* *>* *0.71, *η^2^_p_*s ≦ 0.01; Fig. 8).

### MVPA

**Figure 9.**
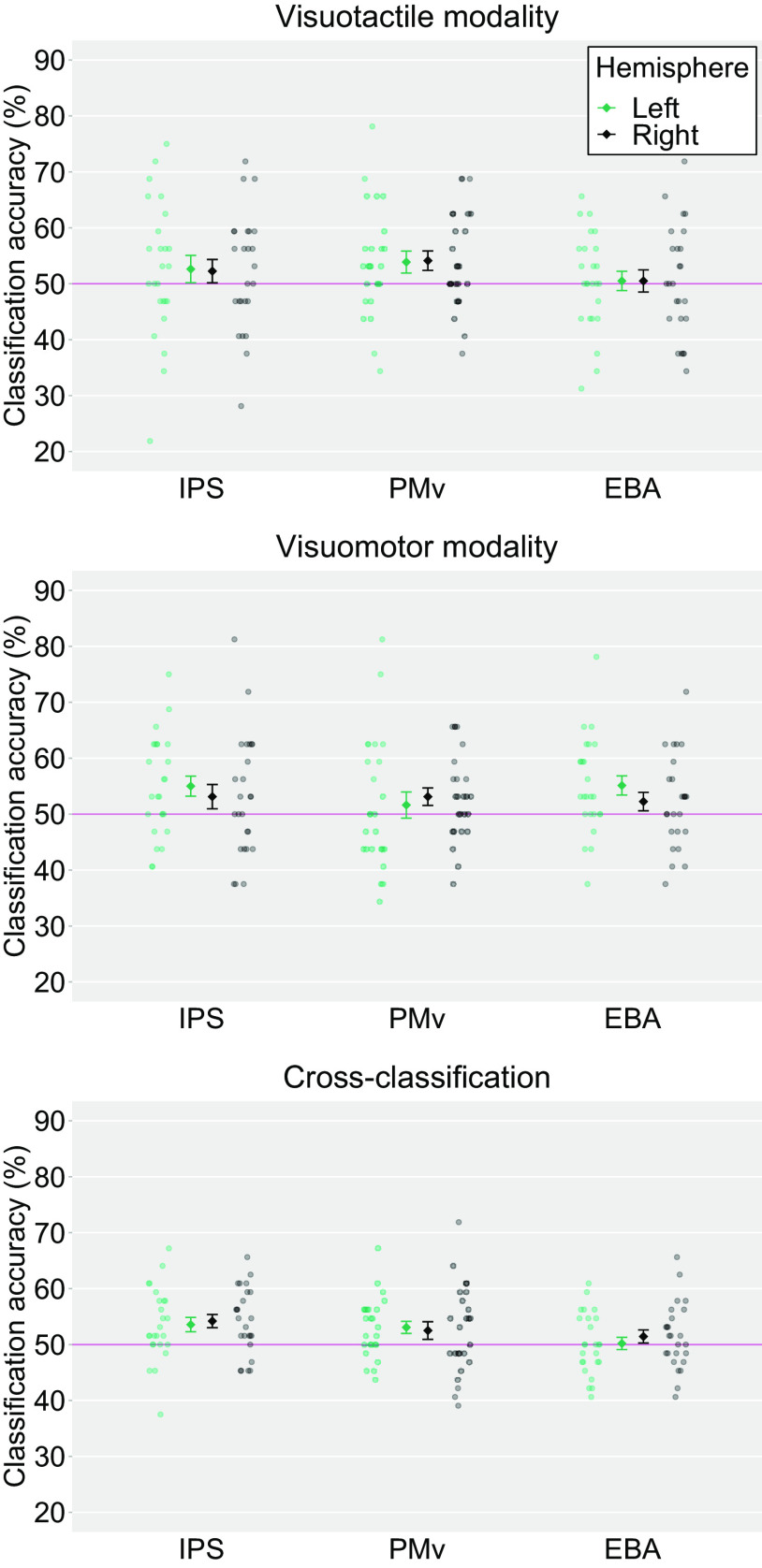
Averaged classification accuracies in each ROI. The light-colored circles correspond to the raw data of each participant (*n *=* *25) and the means are represented as dark-colored markers. Error bars indicate SEM. Horizontal red lines represent chance-level (50%). IPS, intraparietal sulcus; PMv, ventral premotor cortex; EBA, extrastriate body area. Voxel bias of the classifiers are displayed in Figure 10.

We first conducted a ROI-based MVPA to classify the representations of the synchronous and asynchronous conditions within the same and across modalities. Within the visuotactile classification, a significant above-chance decoding accuracy was found for the right PMv (54.12%, *t*_(24)_ = 2.37, *p *=* *0.03, Cohen’s *d *=* *0.48) and a trend toward statistical significance was obtained for the left PMv (53.88%, *t*_(24)_ = 1.98, *p *=* *0.06, Cohen’s *d *=* *0.40). Both these results were not significant after threshold correction for multiple comparisons. Within the visuomotor classification, the classification accuracy was significant for the left IPS (55.00%, *t*_(24)_ = 2.79, *p *=* *0.01, Cohen’s *d *=* *0.57) and the left EBA (55.12%, *t*_(24)_ = 2.98, *p *=* *0.01, Cohen’s *d *=* *0.61). There was a trend toward statistical significance for the decoding accuracy in the right PMv (53.12%, *t*_(24)_ = 2.00, *p *=* *0.06, Cohen’s *d *=* *0.41). Among these ROIs, the decoding accuracies in the left IPS and the left EBA were significant after correction for multiple comparisons. In cross-classification between visuotactile and visuomotor sessions, significant above-chance decoding accuracies were found for the bilateral IPS (53.56%, *t*_(24)_ = 2.77, *p *=* *0.01, Cohen’s *d *=* *0.57 for the left hemisphere and 54.19%, *t*_(24)_ = 3.54, *p *<* *0.01, Cohen’s *d *=* *0.72 for the right hemisphere) and the left PMv (53.06%, *t*_(24)_ = 2.83, *p *=* *0.01, Cohen’s *d *=* *0.58), and the three ROIs showed significant above-chance accuracy after correction for multiple comparisons (Fig. 9).

**Figure 10.**
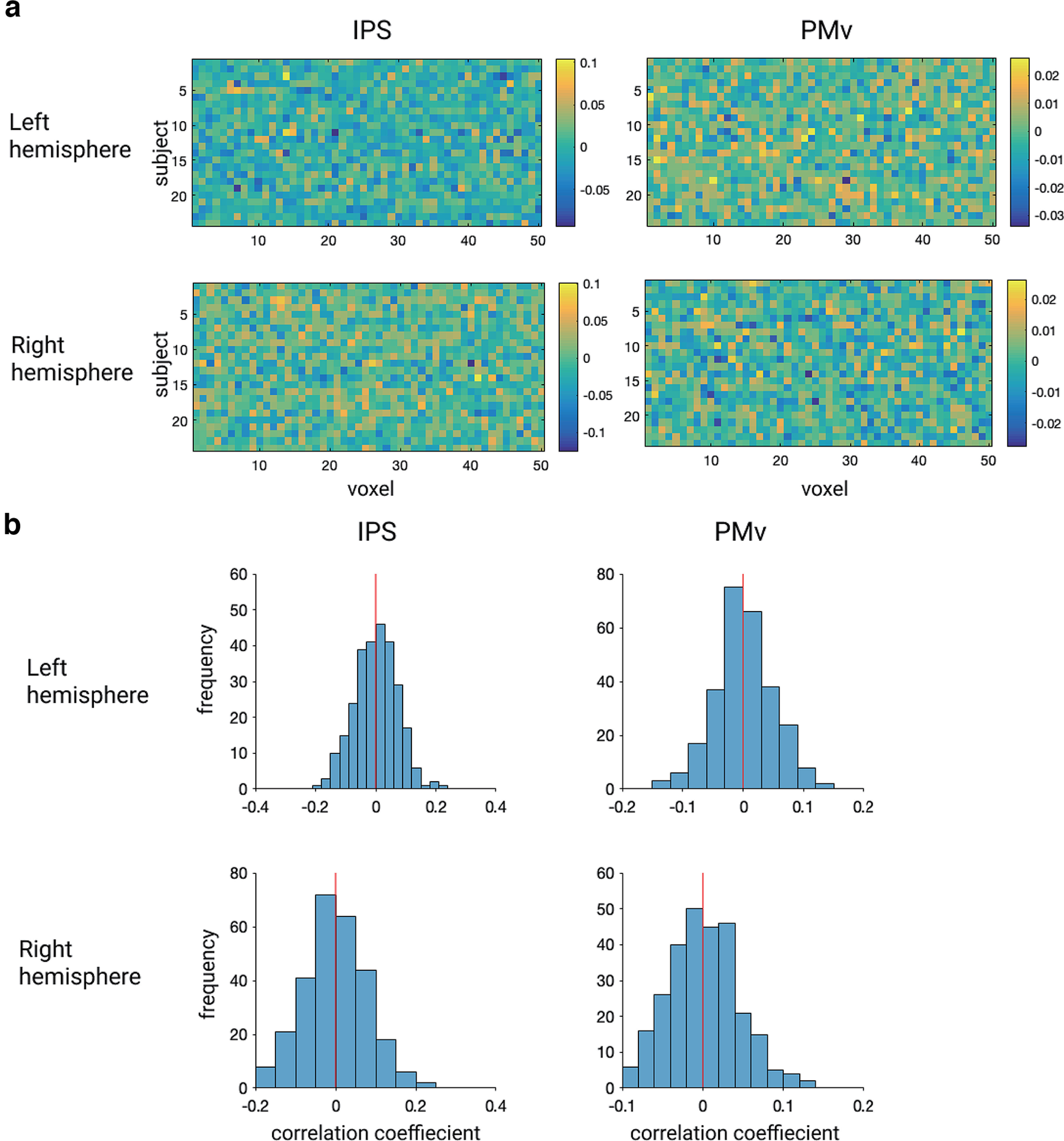
Voxel bias of classifiers. ***a***, Voxel bias maps of “cross-classification” within the ROIs with significant accuracy of timings of visual feedback. ***b***, Distributions of correlation coefficients (R) of bias between different pairs of subjects. IPS, intraparietal sulcus; PMv, ventral premotor cortex.

Additionally, a voxel bias map was generated to investigate whether the neural patterns decoded by MVPA were similar among participants. The voxel bias map displays the average weights (positive or negative) of the classifier across two folds (i.e., iterations) in the cross-validation for individual voxels within ROIs. This map showed intermingled patterns of voxels with two timings of visual feedback (synchronous vs asynchronous biased voxels) within ROIs in all subjects (Fig. 10*a*). In addition, the bias patterns between different pairs of participants revealed low correlations around zero (Fig. 10*b*), indicating idiosyncratic patterns of weights specific to each subject.

**Figure 11.**
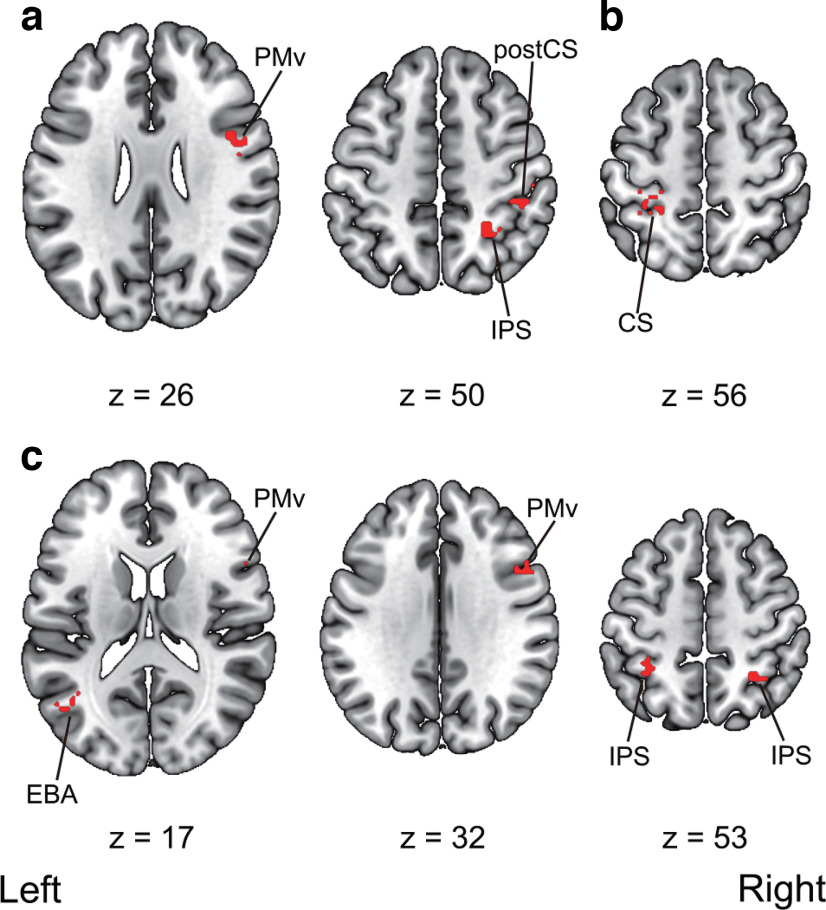
Multivoxel pattern analysis searchlight results. ***a***, Regions that showed significant above-chance decoding accuracy within the visuotactile classification. ***b***, Regions that showed significant above-chance decoding accuracy within the visuomotor classification. ***c***, Regions that showed significant above-chance cross-classification accuracy between the visuotactile and visuomotor classification. Activation was reported with an uncorrected threshold of *p *<* *0.001 at the voxel-level and a threshold of *p *<* *0.05 family-wise error (FWE) corrected at the cluster-level. Montreal Neurologic Institute (MNI) coordinates of the activated foci are reported in Table 6. PMv, ventral premotor cortex; IPS, intraparietal sulcus; postCS, postcentral sulcus; CS, central sulcus; EBA, extrastriate body area. The unthresholded raw *t*-value maps in NIfTY format are available at https://doi.org/10.6084/m9.figshare.19228050.

We then ran searchlight analyses to decode the timings of the visual feedback (i.e., synchronous vs asynchronous) within the same and across modalities. First, significantly above-chance classification accuracies were found in the right IPS, the right PMv, and the right postcentral sulcus within visuotactile sessions. Second, we also observed a significant above-chance classification accuracy in the left central sulcus within-visuomotor sessions. Third, the analysis revealed significant above-chance classification accuracies in the right PMv, the left EBA, and the bilateral IPS in cross-classification between visuotactile and visuomotor sessions. The anatomic locations of the activated areas are reported in Table 6 and Figure 11.

**Table 6:** Anatomical regions, peak voxel coordinates, and *t* values of observed activation of the searchlight multivoxel pattern analysis

Anatomic region	Voxels	MNI coordinates			*t* value
		*x*	*y*	*z*	
Visuotactile classification					
R intraparietal sulcus	27	27	−49	53	5.40
R intraparietal sulcus		36	−43	53	3.84
R ventral premotor cortex	24	48	2	26	5.22
R postcentral sulcus	14	39	−34	50	4.30
R postcentral sulcus		48	−34	50	4.20
R postcentral sulcus		48	−28	44	4.11
Visuomotor classification					
L central sulcus	24	−27	−34	56	4.75
L postcentral gyrus		−33	−31	50	4.39
L postcentral sulcus		−33	−40	59	3.84
Cross-classification					
R ventral premotor cortex	38	51	14	32	5.40
R ventral premotor cortex		51	17	20	4.96
R ventral premotor cortex		45	11	41	4.54
L middle temporal gyrus (EBA)	10	−42	−64	17	5.13
R intraparietal sulcus	22	30	−52	53	4.84
R intraparietal sulcus		30	−49	62	4.12
L intraparietal sulcus	24	−39	−43	59	4.63
L intraparietal sulcus		−33	−46	53	4.18
L intraparietal sulcus		−36	−49	44	3.94

Activation was reported with an uncorrected threshold of *p *<* *0.001 at the voxel-level and a threshold of *p *<* *0.05 family-wise error (FWE) corrected at the cluster-level. MNI, Montreal Neurologic Institute; L, left hemisphere; R, right hemisphere.

### Correlations between cross-classification accuracy and subjective rating

**Figure 12.**
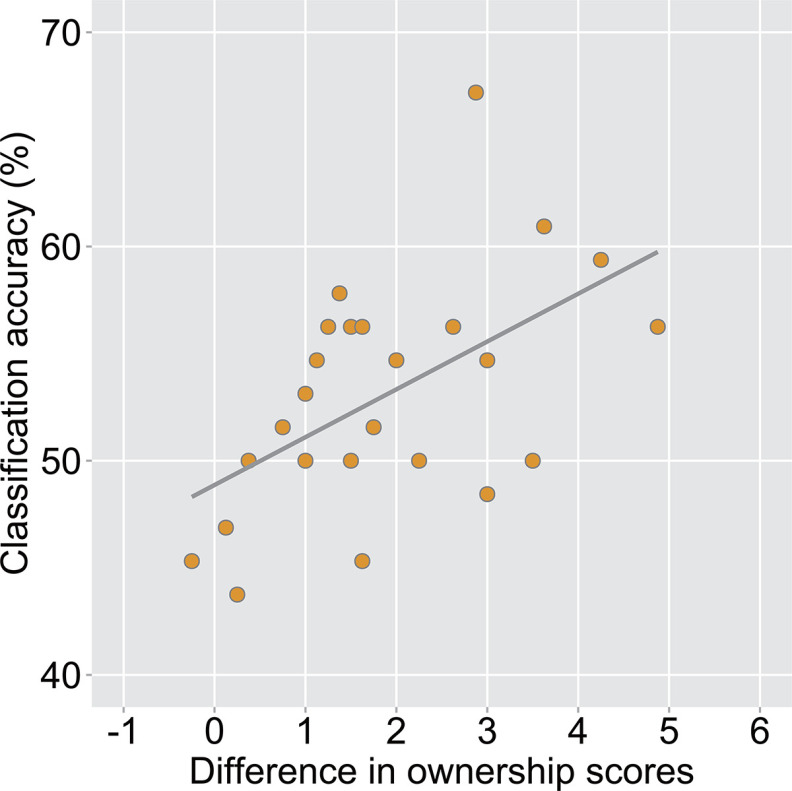
Correlation analysis between the cross-classification accuracy in the left ventral premotor cortex (PMv) and the difference in subjective ratings between the synchronous and asynchronous conditions (Pearson’s *r *=* *0.54, *n *=* *25, *p *=* *0.01). Each circle corresponds to the data of each participant (*n *=* *25).

We analyzed the relationship between the subjective ratings in the questionnaire and the cross-classification accuracies. We first averaged the responses to items (1)–(4) in the visuotactile synchronous and visuomotor synchronous condition and in the visuotactile asynchronous and visuomotor asynchronous condition. We then calculated the differences between the average scores obtained in the synchronous condition and those obtained in the asynchronous condition and correlated the values of these differences with the cross-classification accuracies in each ROI. We found a significant positive correlation between the value and the cross-classification accuracy for the left PMv (Pearson’s *r *=* *0.54, *n *=* *25, *p *=* *0.01) and a trend toward statistical significance for the right IPS (Pearson’s *r *=* *0.38, *n *=* *25, *p *=* *0.06). A significant positive correlation was observed for the left PMv after correction for multiple comparisons (Fig. 12).

## Discussion

The present study tried to reveal the supramodal neural representations of the sense of body ownership for visuotactile and visuomotor modalities. We manipulated the sense of body ownership using visuotactile and visuomotor inputs within a single fMRI experiment and assessed whether the classifier first trained on the data from the visuotactile modality could decode the timings of the visual feedback (i.e., synchronous vs asynchronous) on the data from the visuomotor modality and vice versa. This cross-classification analysis revealed that the IPS, PMv, and EBA subserve the neural representations of the sense of body ownership common to the visuotactile and visuomotor modalities. We also found a statistically significant correlation between the cross-classification accuracy in the left PMv and the difference in subjective ratings between the synchronous and asynchronous conditions. These findings indicate that the sense of body ownership is represented regardless of the modalities in the IPS, PMv, and EBA.

The IPS and PMv exhibited significantly higher cross-classification accuracies for visuotactile and visuomotor modalities in ROI-based MVPA. Such high decoding accuracies were further obtained by searchlight MVPA, providing compelling evidence that there are neural patterns of the sense of body ownership invariant to the modalities in the parieto-premotor cortices. Previous studies showed the role of the IPS and PMv in multisensory integration and the sense of body ownership (Ehrsson et al., 2004; Gentile et al., 2015; Limanowski and Blankenburg, 2015, 2016b, 2018; Grivaz et al., 2017). The role of these regions in the sense of body ownership was also suggested by a lesion study (Zeller et al., 2011) and studies using transcranial magnetic stimulation and transcranial direct current stimulation of these regions (Karabanov et al., 2017; Convento et al., 2018; Lira et al., 2018). Extending previous findings, this study highlights the neural networks involved in the supramodal sense of body ownership induced by multisensory information.

Our searchlight analysis revealed above-chance cross-classification accuracy for the left EBA. The EBA is a region of the LOC involved in the visual processing of the human body such as the perception of one’s movement (Astafiev et al., 2004) and mental imagery of the human body (Blanke et al., 2010). The LOC has been reported to represent peripersonal space with respect to the hands (Makin et al., 2007) as the parietal cortices and PMv (Brozzoli et al., 2011, 2012; Grivaz et al., 2017). The LOC activity is modulated predominantly by the seen position of the hand (Makin et al., 2007). Recent neuroimaging research indicated an increased activation in the LOC, especially in the EBA, to the sense of body ownership (Limanowski et al., 2014; Wold et al., 2014; Limanowski and Blankenburg, 2015, 2016b). Our results provide further insight and reveal a supramodal signature of the sense of body ownership across the sensory modalities in the EBA. However, no significant cross-classification accuracy was observed for the EBA in the ROI-based MVPA, which was performed with ROIs defined with the localizer session. This result inconsistency might be because of the difference in the voxels included in ROIs in the ROI-based and searchlight analyses. It is also possible that in EBA, there are some populations of neurons sensitive to the distinction between visual images of body parts and objects, while there are also others related to the multisensory process of supramodal body ownership with different spatial distributions. However, Limanowski and colleagues reported that clusters of EBA activity associated with the sense of body ownership largely overlap with those that activate specifically to visual body parts (Limanowski et al., 2014). This possibility should therefore be tested in future studies.

The univariate analysis revealed that the averaged activities in the bilateral IPS and PMv were significantly greater in the asynchronous condition than those in the synchronous condition. This seems in disagreement with data from previous RHI studies (Ehrsson et al., 2004; Limanowski and Blankenburg, 2015). However, this discrepancy is likely explained by the differences in the experimental settings. Previous studies (Ehrsson et al., 2004; Limanowski and Blankenburg, 2015) used a fake hand instead of a real one. Thus, visual stimuli might not elicit the sense of body ownership by default in participants and experimental manipulation might be required for participants to feel body ownership. In contrast, in the present study, the participants watched their own video-recorded hand. This might trigger the sense of ownership for the hand displayed on the screen. Interestingly, the results of the present study are consistent with those obtained by Tsakiris et al. (2010) using a similar procedure. Furthermore, Gentile et al. (2013), who recorded videos of tactile stimuli applied to the participants’ own hands before the MRI scan and used them as visual stimuli during the MRI scan, reported that averaged activities in the PMv, IPS, and LOC were greater in the synchronous condition like the previous studies using a fake hand (Limanowski and Blankenburg, 2015). Therefore, it is possible that regions such as the IPS, PMv, and EBA represent the extent to which the “default” sense of body ownership for visual stimuli has been updated by experimental manipulations (i.e., synchronous or asynchronous stimulation). The use of a fake hand or recordings of a real hand (Ehrsson et al., 2004; Gentile et al., 2013; Limanowski and Blankenburg, 2015) may result in larger updates in the synchronous condition, while the presentation of a real hand in real-time (Tsakiris et al., 2010) larger updates in the asynchronous condition. A theoretical model explaining RHI from a predictive coding framework (Apps and Tsakiris, 2014) also suggested that these regions are associated with the default state (in the model, called “empirical prior”) of body ownership. This interpretation is consistent with the claim that the sense of body ownership over the fake hand and the (dis)ownership of the real hand have a common neural basis (Ehrsson, 2020).

Although our results revealed the existence of a shared neural representation of the sense of body ownership across the modalities, our behavioral ratings showed that the sense of body ownership was elicited more strongly in the visuotactile condition than it was in the visuomotor condition. This might be caused by the difference in the threshold detection of the visual feedback delay between the visuotactile and visuomotor conditions. In the present study, the sense of body ownership in the visuotactile condition was induced by visual and proprioceptive information, whereas the sense of body ownership in the visuomotor condition was caused by various afferent signals arising from the muscle spindles, tendon organ, and joint receptors (Proske and Gandevia, 2012). Thus, the visuomotor condition elicited not only the sense of body ownership but also the sense of agency that was not induced in the visuotactile condition (Kalckert and Ehrsson, 2014b). Therefore, in the visuomotor condition, the perception of the mismatch between the intention to move the finger and the visual feedback allowed the participants to detect the visual feedback delay and to determine whether the hand on the screen was their own.

Another possible interpretation of the difference in the behavioral ratings comes from the constraints on the experimental design. While we executed enough trials for MVPA, the stimulus presentation was shorter (i.e., 18 s) than that in previous studies (Ehrsson et al., 2004, 2007; Chae et al., 2015; Olivé et al., 2015; Lee and Chae, 2016). Recent behavioral evidence suggested that the ownership sensation for visuomotor RHI takes ∼23 s to emerge (Kalckert and Ehrsson, 2017). Thus, the duration of the stimulus presentation might have been insufficient for the ownership sensation to emerge, especially in the visuomotor condition, leading to a smaller ownership score in the visuomotor condition than that in the visuotactile condition. Future studies are needed to establish whether the sense of body ownership is induced to the same degree and to determine the stimulation duration necessary to elicit the ownership sensation under different conditions.

The current study also showed the lower subjective ratings in the synchronous and asynchronous conditions compared with the previous fMRI study with similar experimental settings (Tsakiris et al., 2010). The result indicates that the participants might not have felt a strong sense of body ownership, even with a visual feedback of their real hand. This might be because of the intrinsic delay that was inevitable when the videos captured by the camera were presented in real-time. Temporal discrepancies between the tactile stimulation of the real hand or finger movements and the visual feedback caused multisensory integration to break down leading to disembodiment (Roel Lesur et al., 2020). The intrinsic delay of ∼400 ms might have hindered the participants from feeling the hand on the screen as their own, even in the synchronous condition. This can be considered as a limitation to the present study. However, two-way within-subject ANOVA revealed a significant difference in the subjective ratings between the synchronous and asynchronous conditions. We also found higher ratings in the synchronous condition for individual questionnaire items (1)–(4). Thus, the participants perceived the contrast of the visual feedback between the synchronous and asynchronous conditions and judged whether the hand on the screen was their own based on the temporal (in)congruency among sensory signals.

The current study has other methodological limitations. As a first limitation, in the visuomotor condition, the participants received tactile stimulation on the palmar side of the index finger when they executed finger movements. Therefore, a tactile stimulation was present in the visuomotor and the visuotactile conditions. Although this might result in the successful cross-classification between the conditions, we considered that it was unlikely for the following reasons. First, the stimulated body part was different between the conditions: the tactile stimulation in the visuotactile condition consisted in stroking the back of the finger with a paintbrush, while that in the visuomotor condition was on the palmer side of the finger. In addition, as self-generated movements have been reported to attenuate sensory feedback (Blakemore et al., 1998), the stimulation in the visuomotor condition was perceived as weaker than that in the visuotactile condition. Therefore, the successful cross-classification cannot be explained only by the tactile inputs and indicates the sense of body ownership regardless of the modalities. As a second limitation, unlike the study by Tsakiris et al. (2010) investigating the neural correlates of the sense of body ownership and the sense of agency with a similar experimental design, our study did not include the items measuring the sense of agency in the questionnaire. This is because the current study aimed to investigate the common neural representation of the sense of body ownership between visuotactile and visuomotor conditions. The lack of items measuring the sense of agency in the questionnaire is a limitation of this study. Future research needs to investigate the sense of agency in visuotactile and visuomotor conditions using corresponding questionnaire items.

In conclusion, the present study investigated the supramodal neural representations of the sense of body ownership induced by different combinations of sensory inputs. We demonstrated a shared neural representation of the sense of body ownership in the IPS, PMv, EBA for visuotactile and visuomotor modalities. Furthermore, we revealed that the cross-classification accuracy in the left PMv significantly positively correlated with the difference in subjective ratings of the sense of body ownership between synchronous and asynchronous conditions. Our findings provide novel insights into the integration of bodily signals mediating the sense of body ownership.

## Synthesis

Reviewing Editor: Alexander Soutschek, Ludwig-Maximilians-Universitat Munchen Decisions are customarily a result of the Reviewing Editor and the peer reviewers coming together and discussing their recommendations until a consensus is reached. When revisions are invited, a fact-based synthesis statement explaining their decision and outlining what is needed to prepare a revision will be listed below. The following reviewer(s) agreed to reveal their identity: Andrea Serino, Jakub Limanowski. Note: If this manuscript was transferred from JNeurosci and a decision was made to accept the manuscript without peer review, a brief statement to this effect will instead be what is listed below.

Reviewer #1

This study implemented in fMRI the two most used paradigms to manipulate bodily self-consciousness, and in particular body ownership, namely visuo-tactile and visuo-motor stimulation. Classification analyses (based on multi voxel pattern analysis) were used to decode common and specific neural activity associated with the different manipulations. Results pointed to a network of brain regions including PMv, IPS and EBA whose activity encoded synchronous pattern of multisensory stimulation across the different modalities, suggesting an amodal representation of one’s own body. Neural decoding in PMv further correlated with subjective judgments.

These results are not surprising and are in line with a significant line of work from different groups linking multisensory stimulation and body ownership to premotor-parietal networks. Said that, the paper is well done, well written, and methodologically sound and rather innovative.

I have a few suggestions to improve the message and reading of the paper.

Roi Selection :

Why a localizer was used for EBA and not for IPS and PMV? I guess it can be the case because while a clear localizer task is accepted for EBA, this is not the case for the other regions, but authors should explain their choice.

Concerning IPS and PMV, I guess the authors might refer to Grivaz et al. metanalysis which provides converging results for the multiple papers cited by the authors (and more) to support the role of these regions in bodily self consciousness and multisensory integration.

Results:

There are no Figure labels, so I might get wrong the correspondence. Said that:

Figure 4: should report not only the results of the interaction between modality and conditions, but also the main effects.

Figure 6: It would be nice to see where these ROIs are also with respect to this analysis.

These regions are reported in Figure 7-1 (why not Figure 8?), resulting from voxel bais map, but it is not clear whether the two analyses result in similar ROIs.

Discussion:

Page 29, lines: 574-580

“especially in the EBA, to the sense of body ownership (Limanowski et al., 2014; Wold et al., 2014; Limanowski and Blankenburg, 2015, 2016a). However, compared with the IPS and PMv, which were the focus of electrophysiological studies in macaques and neuroimaging studies in humans (Graziano et al., 1997; Duhamel et al., 1998; Ehrsson et al., 2004; Avillac et al., 2007; Tsakiris et al., 2010; Gentile et al., 2011; Olivé et al., 2015), the role of the EBA in the sense of body ownership has not been fully clarified”

EBA almost always emerged in studies assessing multisensory bodily processing within the PPS - e.g., Makin et al., Journal of Neuroscience, 2007, Brozzoli et al., Journal of Neuroscience 2010, 2012), consistently with activation in IPS and PMv - see also the metanalyses by Grivaz et al., 2017. So it is not so surprising to find it as emerging in this searchlight analysis. The authors should refer to this literature and these papers.

Last part of page 29, the discussion about the fact the EBA is significant in the searchlight analysis, but not in the localizer-based MVPA. I agree this likely depends on the different voxels included. This might be by chance, as somehow suggested by the authors, or more interestingly because the classifier task revealed populations of neurons in EBA sensitive to visual images of body parts vs. objects, but in EBA there are other populations of neurons, that are more fundamentally multisensory and participate to body ownership. What do the authors think about this?

The discussion about the different results for visuo-tactile and visuo-motor stimulation is interesting, and this is one of the few studies comparing the two modalities of manipulating body ownership. Whether in the visuo-motor condition there is a prevalent agency manipulation elicited is an interesting and open question. It is a pity that in the questionnaire, no agency question was included. The author should mention this as a limitation, and can also refer to the work by Tsakiris, Longo and Haggard (Neuropsychologia, 2010) where they directly compared ownership and agency by manipulating contrasting synchronous vs. asynchronous and active and passive visuo-motor stimulation.

Minor:

Page 29, lines: 567-569

Consistent with previous findings, our study highlights the neural networks involved in the abstract sense of body ownership induced by multisensory information.

Why “abstract” sense of body ownership? Do the authors mean modality-independent?

This is not clear.

## Author Response

Reply to Reviewer #1

We would like to thank the reviewer for the kind and constructive comments enabling us to substantially enrich the methods, data analyses, and interpretation. We have considered all of these comments and suggestions in our revised manuscript. Red colored text in the manuscript indicates modifications. Our item-by-item responses to the reviewer’s comments are as follows:

Comment #1: Why a localizer was used for EBA and not for IPS and PMV? I guess it can be the case because while a clear localizer task is accepted for EBA, this is not the case for the other regions, but authors should explain their choice.

Response: We appreciate Reviewer #1’s comment regarding ROI selection. We followed the previous studies’ procedures as to whether we used a localizer task to define ROIs. The prior studies have repeatedly used a localizer task to determine the EBA because the EBA cannot be defined based on anatomical landmarks (Limanowski et al., 2014; Olivé, 2015; Limanowski and Blankenburg, 2016b). Conversely, no specific functional localizer task has been used to define the IPS and PMv in previous human neuroimaging studies for the sense of body ownership (Gentile et al., 2013; Limanowski and Blankenburg, 2015, 2016a, 2016b, 2018; Lee and Chae, 2016).

Therefore, anatomical templates were used for the IPS and PMv. We modified our description in the Materials and Methods section to clarify why a localizer was used for the EBA but not for the IPS and PMv.(page 16, line 18 - page 17, line 13)

We followed the previous studies’ procedures as to whether we used a localizer task to define the IPS, PMv, and EBA. Previous human neuroimaging studies for the sense of body ownership have used no specific functional localizer task to define the IPS and PMv (Gentile et al., 2013; Limanowski and Blankenburg, 2015, 2016a, 2016b, 2018; Lee and Chae, 2016). Therefore, anatomical templates for the IPS and PMv were used. The bilateral IPS was defined using Anatomy toolbox (Eickhoff et al., 2005), and the bilateral PMv was defined using the Human Motor Area Template (Laboratory for Rehabilitation Neuroscience, http://lrnlab.org/; Mayka et al., 2006) (Figure 3).

-------------------- FIGURE3 ABOUT HERE --------------------

Conversely, the EBA cannot be identified using templates based on anatomical structures. The prior studies have used a localizer task to determine the EBA (Limanowski et al., 2014; Olivé et al., 2015; Limanowski and Blankenburg, 2016b).

Following the procedure used by Olivé et al. (2015), we measured a significant EBA

2

2

activation during the presentation of body part images compared with that elicited by chair images and determined a sphere with a radius of 5 mm centered on MNI coordinates of the group-level analysis with a threshold of p < 0.05 corrected for family-wise error (FWE) with an extent threshold of 10 voxels (MNI coordinates: [−48, −70, 8] for the left hemisphere and [51, −58, 2] for the right hemisphere).

Comment #2: Concerning IPS and PMV, I guess the authors might refer to Grivaz et al.metanalysis which provides converging results for the multiple papers cited by the authors (and more) to support the role of these regions in bodily self consciousness and multisensory integration.

Response: We would like to thank Reviewer #1 for this valuable comment. We havecited the Grivaz et al. 2017 paper in the Materials and Methods section, as well as explained how the IPS and PMv are associated with the sense of body ownership.(page 16, lines 4 - 8)

The finding is confirmed by a meta-analysis of human neuroimaging studies that investigated the relationship between peripersonal space and the sense of body ownership (Grivaz et al., 2017). Grivaz et al. (2017) have shown that the posterior parietal cortex (the bilateral IPS and superior parietal lobule), right PMv, and left anterior insula are involved in the sense of body ownership.

Comment #3: Figure 4: should report not only the results of the interaction between modality and conditions, but also the main effects.

Response: We appreciate Reviewer #1’s insightful suggestion. Based on this, we have added a new figure of the main effects of modalities and timings of visual feedback in the fMRI univariate analysis as follows:

Figure 5:

Regions activated by the main effects of modalities and timings of visual feedback in the fMRI univariate analysis. a, Activated regions in the visuotactile condition

3

3

compared with the visuomotor condition. b, Activated regions in the visuomotor condition compared with the visuotactile condition. c, Activated regions in the synchronous condition compared with the asynchronous condition. d, Activated regions in the asynchronous condition compared with the synchronous condition. For the main effect of visuotactile and visuomotor conditions, activation was reported with a threshold of p < 0.05 corrected for family-wise error (FWE) with an extent threshold of 10 voxels. For the main effect of synchronous and asynchronous conditions, activation was reported with a threshold of p < 0.001 uncorrected for multiple comparisons with an extent threshold of 10 voxels. Montreal Neurological Institute (MNI) coordinates of the activated foci are reported in Table 2 and Table 3. postCG, postcentral gyrus; CS, central sulcus; ITG, inferior temporal gyrus; PMv, ventral premotor cortex; IFG, inferior frontal gyrus; MTG, middle temporal gyrus.

Comment #4: Figure 6: It would be nice to see where these ROIs are also with respect to this analysis. These regions are reported in Figure 7-1 (why not Figure 8?), resulting from voxel bais map, but it is not clear whether the two analyses result in similar ROIs.

Response: We would like to thank Reviewer #1 for this suggestion. Since we had presented the EBA used in the univariate analysis of the parameter estimates and ROI-based multi-voxel pattern analysis in Figure 7, we added new IPS and PMv figures used in the two analyses in Figure 3.

In this study, the IPS was defined using Anatomy 13 toolbox, and the PMv was defined using the Human Motor Area Template 16. The EBA was defined based on the localizer task’s results. The three ROIs were used in the two analyses. We changed Extended Data Figure 7 to Figure 10. Also, the figure numbers have been changed with Figure 3 and Figure 5 added. Please refer to the revised numbers highlighted in yellow in the manuscript.

Figure 3:

ROIs used in the univariate analysis of the parameter estimates and ROI-based multi-voxel pattern analysis. The bilateral intraparietal sulcus was defined using Anatomy 13 toolbox (Eickhoff et al., 2005). The bilateral ventral premotor cortex was defined using the Human Motor Area Template 16 (Laboratory for Rehabilitation Neuroscience,

4

4

http://lrnlab.org/; Mayka et al., 2006). IPS, intraparietal sulcus; PMv, ventral premotor cortex.

Comment #5: Page 29, lines: 574-580 “especially in the EBA, to the sense of body ownership (Limanowski et al., 2014; Wold et al., 2014; Limanowski and Blankenburg, 2015, 2016a). However, compared with the IPS and PMv, which were the focus of electrophysiological studies in macaques and neuroimaging studies in humans(Graziano et al., 1997; Duhamel et al., 1998; Ehrsson et al., 2004; Avillac et al., 2007; Tsakiris et al., 2010; Gentile et al., 2011; Olivé et al., 2015), the role of the EBA in the sense of body ownership has not been fully clarified” EBA almost always emerged in studies assessing multisensory bodily processing within the PPS - e.g., Makin et al., Journal of Neuroscience, 2007, Brozzoli et al., Journal of Neuroscience 2010, 2012), consistently with activation in IPS and PMv - see also the metanalyses by Grivaz et al., 2017. So it is not so surprising to find it as emerging in this searchlight analysis. The authors should refer to this literature and these papers.

Response: We wish to thank Reviewer #1 for this comment on the role of the EBA in the process of multisensorial information around the body. We have read the suggested papers (Makin et al., 2007; Brozzoli et al., 2011; Brozzoli et al., 2012; Grivaz et al., 2017) and modified the relevant paragraph in the Discussion section as follows:

(page 29, lines 7 - 12)

The LOC has been reported to represent peripersonal space with respect to the hands (Makin et al., 2007) as the parietal cortices and PMv (Brozzoli et al., 2011, 2012; Grivaz et al., 2017). The LOC activity is modulated predominantly by the seen position of the hand (Makin et al., 2007). Recent neuroimaging research indicated an increased activation in the LOC, especially in the EBA, to the sense of body ownership (Limanowski et al., 2014; Wold et al., 2014; Limanowski and Blankenburg, 2015, 2016b).

Comment #6: Last part of page 29, the discussion about the fact the EBA is significant in the searchlight analysis, but not in the localizer-based MVPA. I agree this likely depends on the different voxels included. This might be by chance, as somehow suggested by the authors, or more interestingly because the classifier task revealed populations of neurons in EBA sensitive to visual images of body parts vs. objects, but in EBA there are other populations of neurons, that are more fundamentally multisensory and participate to body ownership. What do the authors think about this?

Response: We would like to thank Reviewer #1 for providing insightful interpretations.

Accordingly, we have deleted a portion of the relevant paragraph in the Discussion section (page 28, lines 19 - 20) and added the following:

(page 29, line 18 - page 30, line 2)

It is also possible that in EBA, there are some populations of neurons sensitive to the distinction between visual images of body parts and objects, while there are also others related to the multisensory process of supramodal body ownership with different spatial

5

5

distributions. However, Limanowski and colleagues reported that clusters of EBA activity associated with the sense of body ownership largely overlap with those that activate specifically to visual body parts (Limanowski et al., 2014). This possibility should therefore be tested in future studies.

Comment #7: The discussion about the different results for visuo-tactile and visuo motor stimulation is interesting, and this is one of the few studies comparing the two modalities of manipulating body ownership. Whether in the visuo-motor condition there is a prevalent agency manipulation elicited is an interesting and open question. It is a pity that in the questionnaire, no agency question was included. The author should mention this as a limitation, and can also refer to the work by Tsakiris, Longo and Haggard (Neuropsychologia, 2010) where they directly compared ownership and agency by manipulating contrasting synchronous vs. asynchronous and active and passive visuo-motor stimulation.

Response: We appreciate Reviewer #1’s comment concerning the fact that no agency question was included in the questionnaire. As Reviewer #1 pointed out, our study’s questionnaire only included the items measuring the sense of body ownership. This stems from the difference in the purpose of our study and Tsakiris et al. (2010). While Tsakiris et al. (2010) investigated the neural correlates of the sense of body ownership and the sense of agency, the primary purpose of our study is to reveal the common neural representation of the sense of body ownership between visuotactile and visuomotor conditions. Therefore, we included only the items measuring the sense of body ownership. We acknowledge that no agency question was included in the questionnaire as a limitation of our study. If the items measuring the sense of agency had been included in the questionnaire, we could have examined the sense of agency between both conditions. We added this point and modified the sentences in the same paragraph in the Discussion section as follows:

(page 33, lines 4 - 6)

The current study has other methodological limitations. As a first limitation, in the visuomotor condition, the participants received tactile stimulation on the palmar side of the index finger when they executed finger movements.

(page 33, line 16 - page 34, line 2)

As a second limitation, unlike the study by Tsakiris et al. (2010) investigating the neural correlates of the sense of body ownership and the sense of agency with a similar experimental design, our study did not include the items measuring the sense of agency in the questionnaire. This is because the current study aimed to investigate the common neural representation of the sense of body ownership between visuotactile and visuomotor conditions. The lack of items measuring the sense of agency in the questionnaire is a limitation of this study. Future research needs to investigate the sense of agency in visuotactile and visuomotor conditions using corresponding questionnaire items.

6

6

Minor comments 1: Consistent with previous findings, our study highlights the neural networks involved in the abstract sense of body ownership induced by multisensory information. Why “abstract” sense of body ownership? Do the authors mean modality independent? This is not clear.

Response: We regret that the sentence lacked clarity. We intended to state that our study highlights the neural networks involved in the supramodal sense of body ownership. We modified the sentence in the Discussion section as follows:

(page 29, lines 2 - 3)

Extending previous findings, this study highlights the neural networks involved in the supramodal sense of body ownership induced by multisensory information.
